# Development of a New Approach to Aid in Visual Identification of Murine iPS Colonies Using a Fuzzy Logic Decision Support System

**DOI:** 10.1371/journal.pone.0070605

**Published:** 2013-08-08

**Authors:** Vinicius Bassaneze, Chester Bittencourt Sacramento, Rodolfo Freire, Patrícia Fernandes De Alencar, Neli Regina Siqueira Ortega, Jose Eduardo Krieger

**Affiliations:** 1 Laboratory of Genetics and Molecular Cardiology/LIM 13, Heart Institute (InCor), University of São Paulo Medical School, São Paulo, São Paulo, Brazil; 2 Center of Fuzzy Systems in Health, Faculty of Medicine, University of São Paulo, Department of Pathology, São Paulo, São Paulo, Brazil; University of California, San Diego, United States of America

## Abstract

The *a priori* identification of induced pluripotent stem cells remains a challenge. Being able to quickly identify the most embryonic stem cell-similar induced pluripotent stem cells when validating results could help to reduce costs and save time. In this context, tools based on non-classic logic can be useful in creating aid-systems based on visual criteria. True colonies when viewed at 100x magnification have been found to have the following 3 characteristics: a high degree of border delineation, a more uniform texture, and the absence of a cracked texture. These visual criteria were used for fuzzy logic modeling. We investigated the possibility of predicting the presence of alkaline phosphatase activity, typical of true induced pluripotent stem cell colonies, after 25 individuals, with varying degrees of experience in working with murine iPS cells, categorized the images of 136 colonies based on visual criteria. Intriguingly, the performance evaluation by area under the ROC curve (16 individuals with satisfactory performance), Spearman correlation (all statistically significant), and Cohen's Kappa agreement analysis (all statistically significant) demonstrates that the discriminatory capacity of different evaluators are similar, even those who have never cultivated cells. Thus, we report on a new system to facilitate visual identification of murine- induced pluripotent stem cell colonies that can be useful for staff training and opens the possibility of exploring visual characteristics of induced pluripotent stem cell colonies with their functional peculiarities. The fuzzy model has been integrated as a web-based tool named “2see-iPS” which is freely accessed at http://genetica.incor.usp.br/2seeips/.

## Introduction

Identifying emerging induced pluripotent stem (iPS) cell colonies based solely on morphological criteria is possible [1]; however, it is difficult to classify *a priori* which ones are true, that is, those that will have characteristics similar to the characteristics of embryonic stem (ES) cells after being collected and reprogrammed. About 75 to 88% of the colonies that develop a few days after viral transduction are probably false [2], not having the typical ES molecular marker cells after being collected, possibly due to a defective or an incomplete reprogramming process [3].

Usually the initial identification of potential colonies is performed using reporter gene systems with fluorescent probes or antibiotic resistance for ES pluripotency genes, such as Fbox15 [4], UTF1 [5], [6], Nanog [7], [8], and Oct3/4 [2]. These systems facilitate the rapid identification of true colonies before collection without killing them, unlike assays necessary for verification of expression of alkaline phosphatase (based on regular substrates) or other intracellular molecular markers. However, they can only be used in cells derived from transgenic animals containing these systems, or primary cell cultures previously treated with viruses, to introduce the system into their genome. In addition, sometimes there is a faint expression of these nuclear markers on non-true colonies [9]. To date there is only one method, recently described, capable of detecting alkaline phosphatase positive cells using a fluorogenic substrate without destroying cells [10]. This is a promising approach, however, still involves increasing processing time and costs using reagents which possible negative effects are not fully studied. Altogether, these still may pose a problem when it is necessary to collect several colonies and, subsequently, to validate them as pluripotent, especially when it is not suitable to introduce exogenous elements into genomes, such as human cells that will be used in the future in clinical practice.

The use of alkaline phosphatase has been used as initial indicator of successful iPS cell reprogramming. However, to definitively establish that complete reprogramming has occurred, these colonies must display high similarity to ES cells using other assays. Global gene expression, epigenetic methylation profile, generation of teratomas when injected into nude animals, and the ability to generate fully fertile chimeric animals when injected into a blastocyst (for nonhuman cells) are frequently assayed to assure that a particular cell colony has pluripotency. However, these are very laborious and expensive and significant differences between them are still observed [11]. Therefore, new approaches for accurate screening to select colonies remain of great interest.

It is possible to observe great morphological diversity in iPS cell colonies before picking. The association between the flat morphology in non-pluripotent colonies in contrast to the “ES-like” morphology of true mice iPS colonies has already been observed, particularly when colonies are reprogramed without feeder cells [2]. Recently, it was proposed seven morphological measurable parameters that can be used to qualify human iPS cells [12]. In an attempt to reduce work-time and the cost of collecting false colonies, we developed a test using 3 visual parameters as the first element in decision-making for picking true mice iPSCs colonies.

## Materials and Methods

### Mouse embryonic fibroblast extraction and culturing

All procedures were approved by the University of São Paulo General Hospital Ethics Committee (Protocol #0949/08). Mouse embryonic fibroblasts (MEF) were used as feeder cells and for cell reprogramming. These cells were obtained based on a protocol described previously [13]. In summary, Swiss mouse embryos [14] (14 days post coitus) had yolk sacs and livers removed and then fragmented into small pieces with the aid of scissors. The fragments were incubated in digestion buffer (DMEM low glucose supplemented with 0.25% porcine pancreas trypsin and 0.02% EDTA, 5 mL per embryo) at 37°C for 45 minutes. Then the solution was left to decant, and the remaining cells of supernatant were platted in T.150 bottles (one embryo per bottle), with 20 mL of MEF culture medium (DMEM low glucose (Invitrogen) supplemented with 15% bovine calf serum (BCS, Hyclone) and 1% L-glutamine and 1% P/S (Invitrogen). After 24 hours, it was possible to observe adhered cells. For iPS generation, cells were used on passage 2. To obtain feeder cells, the cells were expanded every 2–3 days until passage 3, when they had proliferation inhibited with mitomycin C (12 µg/mL for 3 h) [15].

### Generation of iPS colonies

MEF were reprogrammed into iPS cells by using the method developed by Yamanaka and colleagues [13] with minor modifications. First, retroviruses were produced in HEK-293T cells (293T) transfected, separately, with the pMX retroviral vectors containing the murine sequence for each of the necessary genes for reprogramming: Oct3/4, Sox2, Klf4, and c-Myc (Addgene, Cambridge, MA). pMX-eGFP viruses were also produced (built in the laboratory) and served as an indicator of the efficiency of retroviral transduction. Once the efficiency of virus production was high (at least 70–80%), MEF in passage 2 were plated in 60-mm gelatinized plates at a concentration of 1×10^6^ cells, and infected with 500 μL of supernatant from each of the 4 or 3 (except cMYC) reprograming viruses in the presence of 8 μg/mL polybrene (Sigma). After 5 days, cells were trypsinized, and 5×10^4^ cells were plated in a new P100 plate previously containing 8×10^5^ feeder cells. The next day, cell medium was changed to iPS medium (DMEM High Glucose supplemented with 15% Knockout serum, 1% MEM nonessential amino acids, 1% L-glutamine, and 10ng/mL murine leukemia inhibitory factor (LIF), 0.1 mM β-mercaptoethanol, 50 U/mL P/S [all Invitrogen]). After 1 week, cell colonies began to emerge, which were followed for up to 30 days when pictures were taken to create an image database. After this period, some colonies were transferred to plates for further expansion, frozen in stock, and all had alkaline phosphatase activity checked.

### Phosphatase alkaline activity

The assay for determination of alkaline phosphatase activity was performed in 24 gelatinized well plates following manufacturer's instructions (Leukocyte Alkaline Phosphatase Kit, Sigma).

### Visual interpretation

Several variables could be evaluated to visually characterize colonies, including some related to image properties, which were further used to create an automated computer assessment. Here, as proof of concept, the images chosen displayed characteristics described using human vocabulary. Namely, the degree of (1) border delineation, (2) texture uniformity, and (3) cracked texture were used in an almost linear fashion to create the fuzzy model. We also noted that colony size, nucleus/cytoplasm ratio (under higher microscope magnifications), and time after the infection procedure were also very important, but they were not included, in order to facilitate the creation of a visual tool, regardless of the level of staff specialization.

In this study, the definition of a true colony was based on having positive staining for alkaline phosphatase after 30 days of viral infection and the capacity to proliferate after picking, all duly noted and omitted from all observers. Altogether, 136 images were evaluated by 25 different individuals with different expertise. Five were defined as “specialists” (individuals who work directly with this cell type and already had experience with nuclear reprogramming experiments), nine “practical” persons (already had practice with cell culture, but not with iPS/ES cells), five “nonpractical” persons (work in a biomedical laboratory but had no practice with cell culture), and finally six laypersons, without laboratory experience. All received written instructions and had to evaluate all images to determine the degree varying from 1 to 5 of those 3 visual characteristic (**Figure S1**), compared to a reference guide. In addition, specialists were also challenged to categorize all images as true or false based on their preconception. These data were used to perform ROC and Cohen's Kappa agreement analysis.

### Fuzzy logic modeling

In the fuzzy model, rankings varying from 1 to 5 for each variable from each image were linearly normalized to fuzzy degrees of membership ranging from 0 to 1. Three membership functions of triangular shape were used to input data (**Figure S2**). All procedures were performed using Matlab 2010 and Microsoft Excel 2010.

Furthermore, the rules database that composes the linguistic model was created from these membership functions, which establish a connection between the variables and membership functions previously stated (**Figure S3**). The fuzzy model was integrated as a web-based tool named “2see-iPS,” which is freely accessed and currently in five different languages at http://genetica.incor.usp.br/2seeips/.

### Model performance evaluation

Two forms of performance evaluation were used: measure of model accuracy from the ROC curve, and Cohen's Kappa agreement analysis to assess the ability to identify the cells using the model compared to human experts. Model output of expert compared to non-expert evaluators was verified by ROC curve. Correlation between the model outputs was determined using the Spearman correlation, comparing all kind of expertise, from experts to laypeople. The same statistic method was applied for examination of the uniformity of three inputs (border delineation, texture uniformity and cracked texture) among the different groups of observers, however, correlating the consensus (median) of the specialists to the other groups. The ROC (Receiver Operating Characteristic) is a powerful tool to express the relationship between true-positives (sensitivity) and false-positives (one minus specificity). The area under the curve is associated with the performance classification and varies from 0.5 to 1. Indexes above 0.7 are considered “satisfactory”. The optimal threshold value was established from ROC curve data by calculating the average of the highest product, for each observer, of sensitivity per specificity. For better data visualization, fuzzy model output was categorized in positive, negative, false positive and false negative, for each observer and colony, and was graphically clustered using centroid linkage and city-block distance similarity metric using Cluster Gene 3.0 and JavaTreeView software.

Cohen's Kappa agreement was performed between assessment of true and false colonies (assayed by phosphatase alkaline) and 5 specialists' preconception judgment. When statistically significant, its ranges from 0 to 1, and the interpretation is given by the following ranges: bellow 0 no agreement, 0 to 0.19 poor agreement, 0.20 to 0.39 fair agreement, 0.40 to 0.59 moderate agreement, 0.60 to 0.79 good agreement, and 0.80 to 1.00 very good agreement [16]. All tests were performed using Microsoft Excel 2010 and SPSS Statistics 19.0 software. Differences between colonies sizes were analyzed using w way ANOVA with Bonferroni post hoc test. A level of 5% was considered statistically significant.

## Results

### Colony image characteristics but not colony size can discriminate between true and false iPS colonies

**Figure 1:**
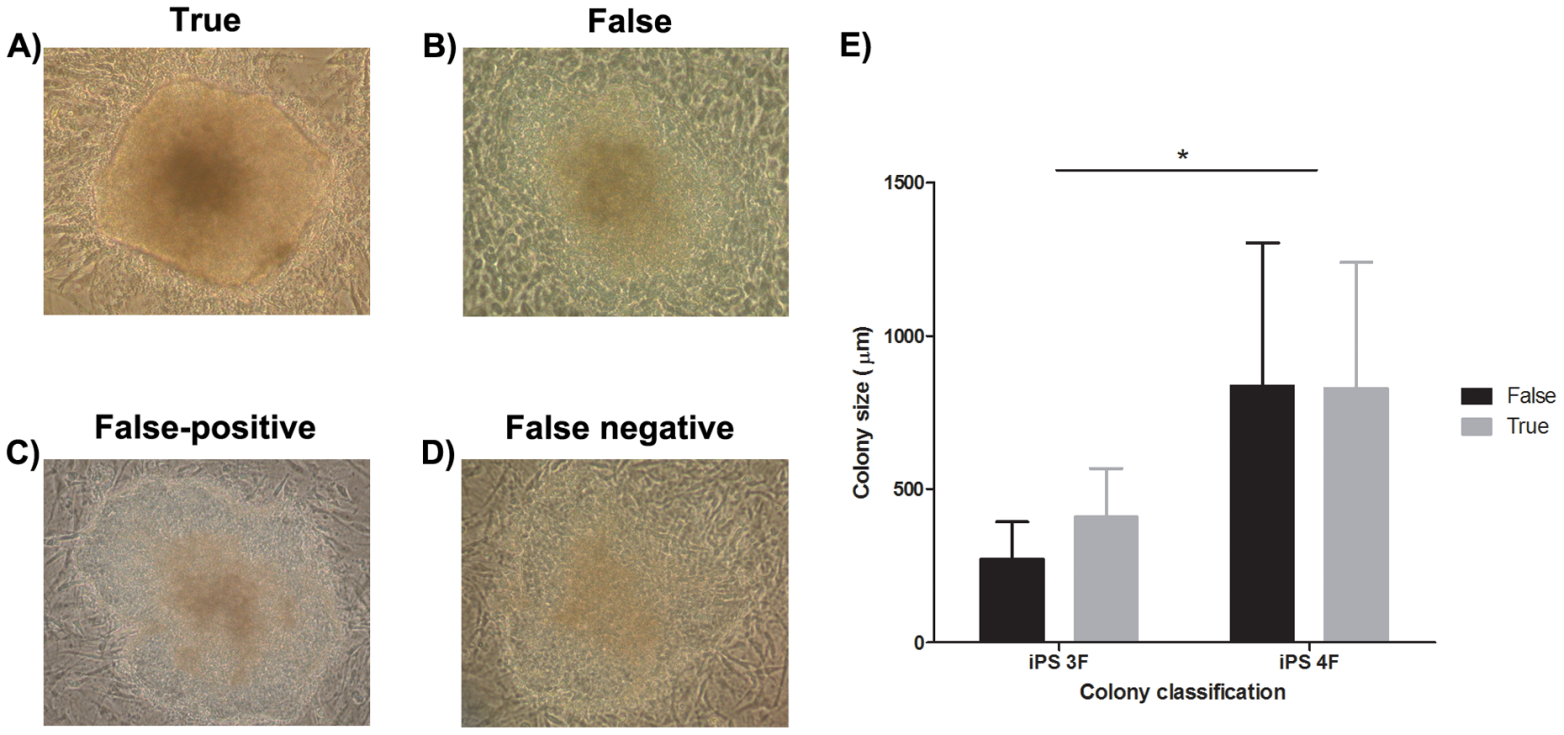
Colony's image characteristics. Representative images of (**A**) true, (**B**) false, (**C**) false-positive and (**D**) false-negative iPS colonies having approximately the same size on day 30 (100x augmentation). (**E**) iPS Colony size and number of nuclear factors were investigated to determine if they were capable of predicting true iPS; however, only a non-statistical trend was observed; (mean ± SD, 2W ANOVA, * indicates P<0.05).

One hundred and third-six colonies were generated, photographed before picking, and assayed for alkaline phosphatase activity. A wide variety of size and morphology colonies can be observed 30 days after viral infection **(** **Fig 1** **A–D)**. Images were grouped according to alkaline phosphatase activity into 2 groups, positive (60.3%) and negative (39.7%) colonies, which hereafter will be called true and false iPS colonies. Three image characteristics were related to true colonies under 100x magnification: a high degree of border delineation, a more uniform texture, and the lack of cracked texture. Colony size was also determined and, in spite of the expected cMyc effect, there was no association with its false/true categorization (**Fig** **1E**; Two-way ANOVA, p>0.05). In addition to alkaline phosphatase, for some colonies we also checked iPSCs molecular markers (RT-PCR and immunofluorescence), performed teratoma formation and generated chimera embryos **(Figure S4** **and Material and Methods S1).**

### Specialists can correctly discriminate true colonies based on preconception judgments

To verify the agreement between the assessment of true and false colonies (assayed by phosphatase alkaline) and 5 specialists' preconception judgments, Cohen's Kappa analysis was performed (**Table** **1**), without using the fuzzy model system. Although in one case (Specialist 5) the agreement was considered poor, most of the time there was substantial or moderate agreement, including in the proof. The average sensitivity, specificity, false positive and false negative rates were, respectively, 67.6%±22.2%, 77.4%±11.2%, 9%±4.5 and 19.6%±13.4%. The average hit rate was 71.47%.

**Table 1:** Specialists were asked for classify colonies based on their own perception.

Kappa	Proof	Specialist 2	Specialist 3	Specialist 4	Specialist 5
**Specia list 1**	0,664	0,588	0,511	0,366	0,315
**Specia list 2**	0,486	–	0,506	0,373	0,246
**Specia list 3**	0,54	–	–	0,273	0,177
**Specia list 4**	0,315	–	–	–	0,393
**Specia list 5**	0,187	–	–	–	–

Cohen's Kappa agreement analysis of prejudgment of colony status. Values ranging from 0 to 0.19 were considered poor agreement, 0.20 to 0.39 satisfactory, 0.40 to 0.59 moderate, 0.60 to 0.79 substantial and 0.80 to 1.00 almost perfect agreement.

### The colonies characteristics score assignment does not vary significantly among evaluators with different expertise on iPS cells

**Figure 2:**
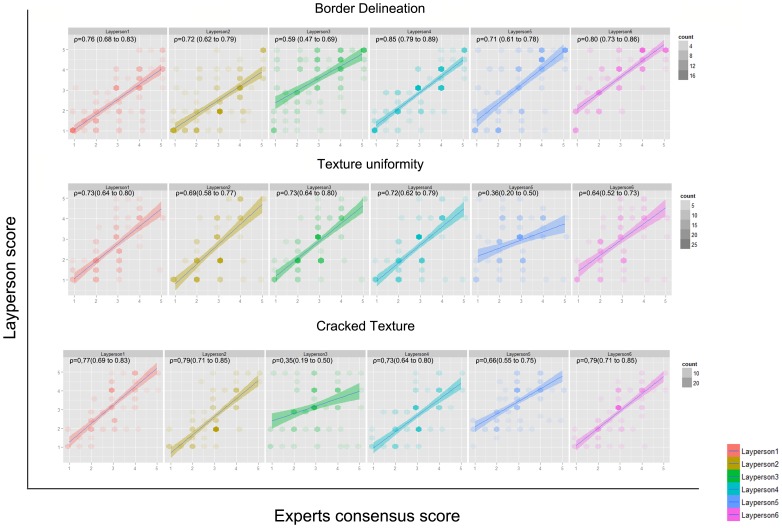
Score's assignment to colonies. Comparison between laypeople and expert consensus for colonies score degree of border delineation, texture uniformity and true cracked texture. (Spearman rho correlation (95% confidence interval, all significant non-zero, P<0.001). Except layperson 3 (cracked texture) and 5 (texture uniformity), all had good correspondence to experts' image perception.

To verify the hypothesis that individuals with different expertise have dissimilar assessment profiles, we compared their assigned scores for the 3 image characteristics considered by each group of observers. Unexpectedly, the discriminatory capacity by different layman evaluators does not vary significantly compared to specialists (**Fig** **2** **,** spearman rho correlation (95% confidence interval), all significant non-zero, P<0.001). Except layperson 3 (cracked texture) and 5 (texture uniformity), all had good correspondence to experts' image perception. The same can be concluded for practical and non-practical groups (data not shown). This indicates that neither experience with this cell type nor cell culture practices are a prerequisite for image feature extraction, after proper instructions.

### Evaluation of 3 image characteristics is sufficient to significantly increase the correct identification of true colonies

**Figure 3:**
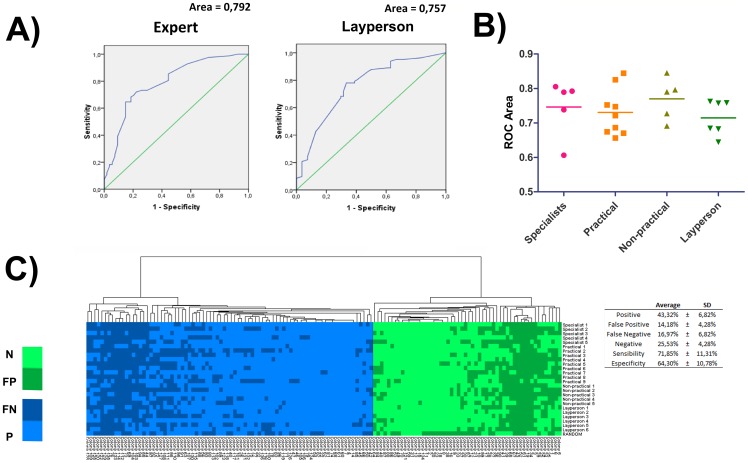
Fuzzy model performance evaluation. (**A**) Representative ROC curves showing the relationship between true- and false-positives when varying the decision criterion. Note that the profile of experts and layperson can be very similar (**B**) Corresponding area under the ROC curve that is associated with the classification performance among different individuals. Sixteen individuals had ROC area above 0.7, categorizing it as a satisfactory performance. (**C**) Graphical representation of true positive, true negative, false positive and false negative classification of all colonies studied for each observer. Data was clustered in order to ease the visualization.

**Figure 4:**
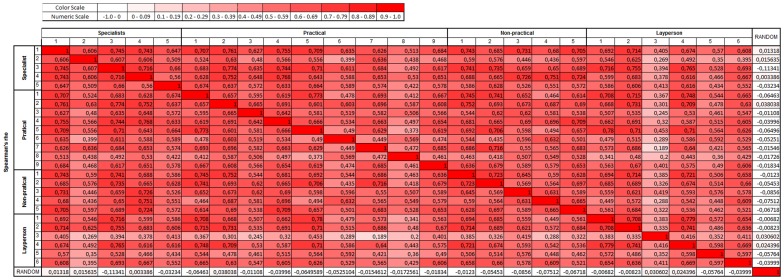
Correlation analysis by Spearman method of fuzzy model output. All red colored cells are considered statistically significant (p<0.01).

Collected data were processed in a fuzzy logic-based system, and a criterion cutoff was established for the output to categorize the colonies between true or false. ROC curves clearly demonstrated that there was satisfactory performance on classification, except for 9 individuals (ROC area under the curve below 0.7 for Specialist 2, Practical 1, 3, 5 and 9, Non-practical 2 and Layperson 1, 3 and 5; **Fig** **3A**). However, all the group means are above this threshold. It was possible to evaluate the general performance of the 25 observers to classify the current status of each colony using the fuzzy system and an average cutoff common to all (**Fig** **3B** **;** positive, negative, false positive and false negative status, graphically clustered). It is interesting to note that several colonies were correctly assigned as negative and positive by all observers using the system, despite their experience status. The average sensitivity, specificity, false positive and false negative rates were, respectively, 71.9%±11.3%, 64.3%±10.8%, 14.2%±4.3 and 17.0%±6.8% (mean ± SD, **Fig** **3C**). The average hit rate was 68.85%.In addition, the Spearman correlation was statistically significant for all comparisons, including those among different specialists and layperson (**Figure** **4**). That indicates that all individuals provided a set of responses for all 136 images that was compatible with each other using fuzzy logic, including Specialist 5, that did not had a good performance on prejudging true colonies. Except for layperson 3, who had a slightly lower performance than the others (even though statistically different from random), all seem to use the same criteria for classification, acquiring a significantly similar answer tendency.

Taken together, these results strongly suggest that the fuzzy system design is able to assist in the classification of murine iPS colonies with performance equal to or greater than the visual classification performed by individuals with direct experience in the identification of true iPS colonies.

## Discussion

In this article, we present the results of the generation of a system to aid the identification of true induced pluripotent cell colonies using fuzzy logic. Based solely on visual criteria, it is possible to predict with a non-negligible accuracy the phosphatase alkaline activity status of iPS cells.

Given the difficulty of visual classification of the colonies due to its subjectivity, an interesting way to approach this problem, among others in the field of artificial intelligence, is using systems based on fuzzy logic. The fuzzy sets theory was created initially by Lotfi A. Zadeh in 1965 [17] and is particularly useful when it is difficult to classify whether an element should or should not belong to a given group of classical binary logic. To deal with this uncertainty, he created a new concept called the “degree of membership” with an element, which could belong partially to a given set. This degree can vary from zero to one, where zero indicates that the element does not participate in the group; one indicates that an element participates fully in the group; and the intermediate value of the element gives the possibility to participate in part of the group. From this theory, it is possible to model and manipulate vague and imprecise information through linguistic rules based on expert knowledge. This theory has been used successfully in the generation of mathematical models that have been used in practical applications in various fields of knowledge [18], particularly in process control where there is need for decision making [19].

The use of fuzzy logic for image processing even in the field of cytology is not uncommon. Recently, Lin at al created a fuzzy inference system to identify in-vitro colonies of cancer cells [20], and a several unpublished works can be found that use fuzzy logic for cell image segmentation[21]. Using the fuzzy sets theory as a tool to classify murine iPS colonies seems effective. This is quite interesting because it is not uncommon in the literature to discredit the possibility of visually distinguishing true from false colonies, except when there are large morphological differences between them and in the absence of a feeder layer.

Although this work was not designed to evaluate all parameters to define pluripotency of a colony, the evaluation of alkaline phosphatase is an important step and performed in almost all laboratories. In contrast to the readiness and ease of use of alkaline phosphatase assay, it has been reported a significant false positive rate [22] for this staining (considering as gold standard to demonstrate bona fide iPS cells). We speculate that this might be, at least in part, due to the subjective observation of a non-uniform staining (as reported for human iPS cells, [23]) as a result of faintly (or partially) stained colonies, probably false ones, or indeed non reprogramed cells. This new approach may be useful for at least 2 goals: reducing costs and effort in the collection of colonies. This can be valuable in situations where it is necessary to work with cells without genetic modifications to large-scale production. Moreover, this approach can be adapted to be used for the training of laboratory personnel to visualize the correct colonies. Even without using size and time, the results are satisfactory suggesting that these are the main variables for this identification.

Thus, the new system reported herein, as proof-of-concept, can facilitate visual identification of murine iPS colonies, which can be useful for staff training and opens the possibility of exploring visual characteristics of iPS colonies with their functional peculiarities. The fuzzy model was integrated as a tool named “2see-iPS” which is freely accessed at http://genetica.incor.usp.br/2seeips/.

## Supporting Information

Figure S1 **Images used as a guide for colony classification, in a linear fashion, by all observers.** If there were doubts about the classification between 2 images, subjects were instructed to note the median value (e.g. 3.5 when in doubt between 3 or 4). Images were intentionally left in grayscale, small-windowed, and softly blurred to highlight the variable to be determined (e.g. nonhomogeneous images can be cracked).
(TIF)

Figure S2 **Membership functions of the fuzzy sets.** The scores for colony's (**A**) border definition degree and texture homogeneity degree have membership functions given by the predicates “Low” (from 0 to 0.5), “Medium” (0.0–1) and “High” (0.5–1). (**B**) Cracked texture has the predicates “Absent” (from 0 to 0.5), “Possible” (0.0 to 1) and “Present” (0.5 to 1). As output, after defuzzyfication, there was “not IPS cells”, “probably non-iPS”, “Undefined” “probably iPS” or “iPS” domains. The optimal threshold value to dicotomically classifies between true and false colonies were calculated as 0.434. As an example, the true iPS colony on Figure 1 can be categorized as 0.875 for texture homogeneity and border delineation and 1 for cracked texture. After the defuzzyfication, 0.72 emerges as output and the colony is classified as true.
(TIF)

Figure S3 **Fuzzy logic control surface output.** There is a non-linear variation of the possibilities for fuzzy output depending on the observed degree of texture uniformity and (A) border delineation or (B) cracked texture.
(TIF)

Figure S4 **iPS generation using retrovirus.** (**A**) Morphology of a positive iPS cell after picking (**B**) representative picture of alkaline phosphatase activity after picking (100x magnification). (**C**) Verification of pluripotency markers by RT-PCR. (**D**) Visualization by fluorescence microscopy of SSEA1 iPS cells marker. (**E**) Verification of the 3 germ layers formation on teratoma structures in nude animals. (**F**) Chimerical mouse fetus (in E10) viewed under 20x magnification (right filter for GFP). It is possible to see that virtually the whole body of the embryo is fluorescent, including the heart and the developing members, but not the placenta (non-iPS origin).
(TIF)

Materials and Methods S1 (DOCX)
