# Supplementary figures and images for: Development of a New Approach to Aid in Visual Identification of Murine iPS Colonies Using a Fuzzy Logic Decision Support System
Source: PLoS One. 2013 Aug 8;8(8):e70605. doi: 10.1371/journal.pone.0070605 (PMC3738584; doi:10.1371/journal.pone.0070605)

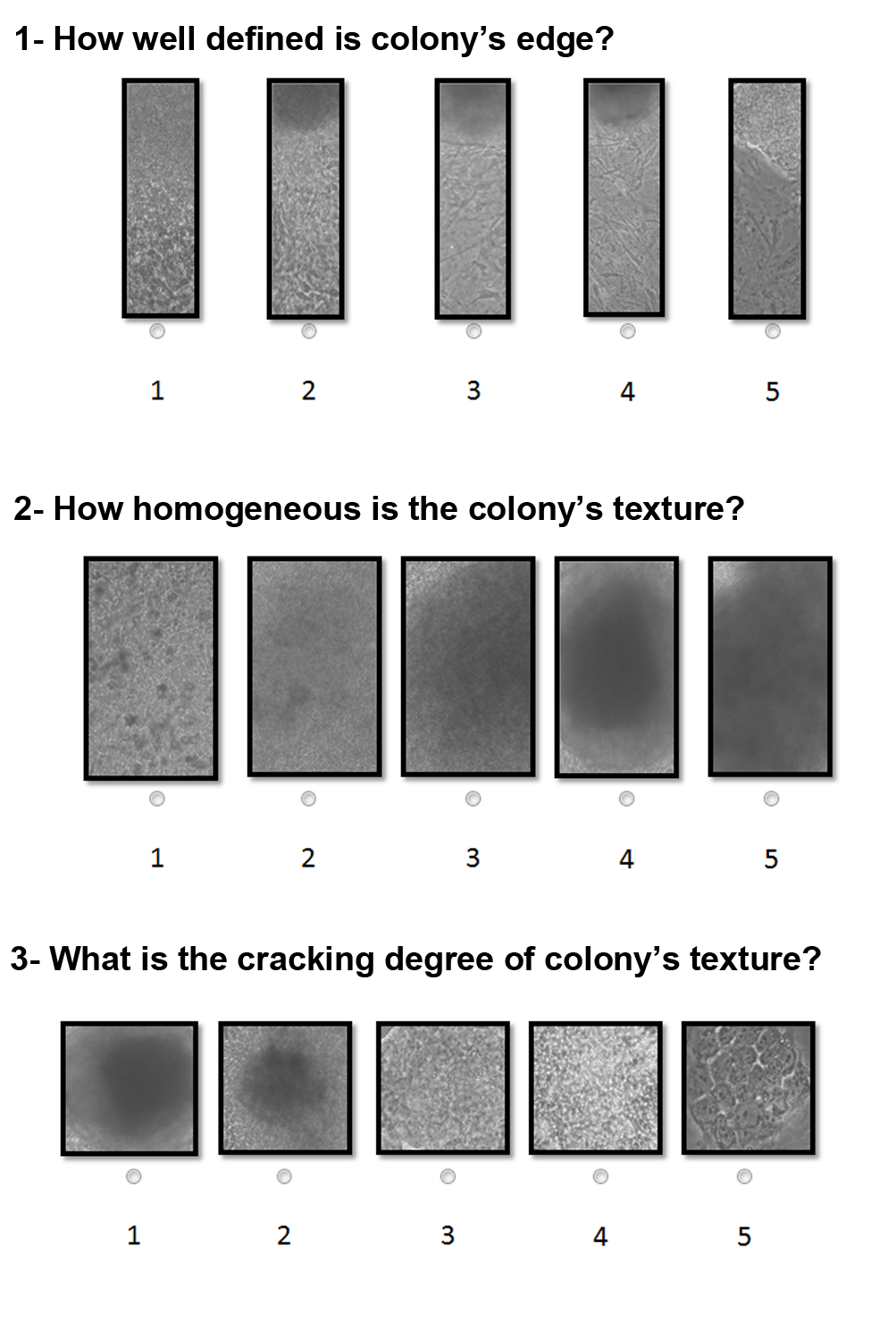

Supplement: Figure S1 — Images used as a guide for colony classification, in a linear fashion, by all observers. If there were doubts about the classification between 2 images, subjects were instructed to note the median value (e.g. 3.5 when in doubt between 3 or 4). Images were intentionally left in grayscale, small-windowed, and softly blurred to highlight the variable to be determined (e.g. nonhomogeneous images can be cracked). (TIF) [file pone.0070605.s001.tif]

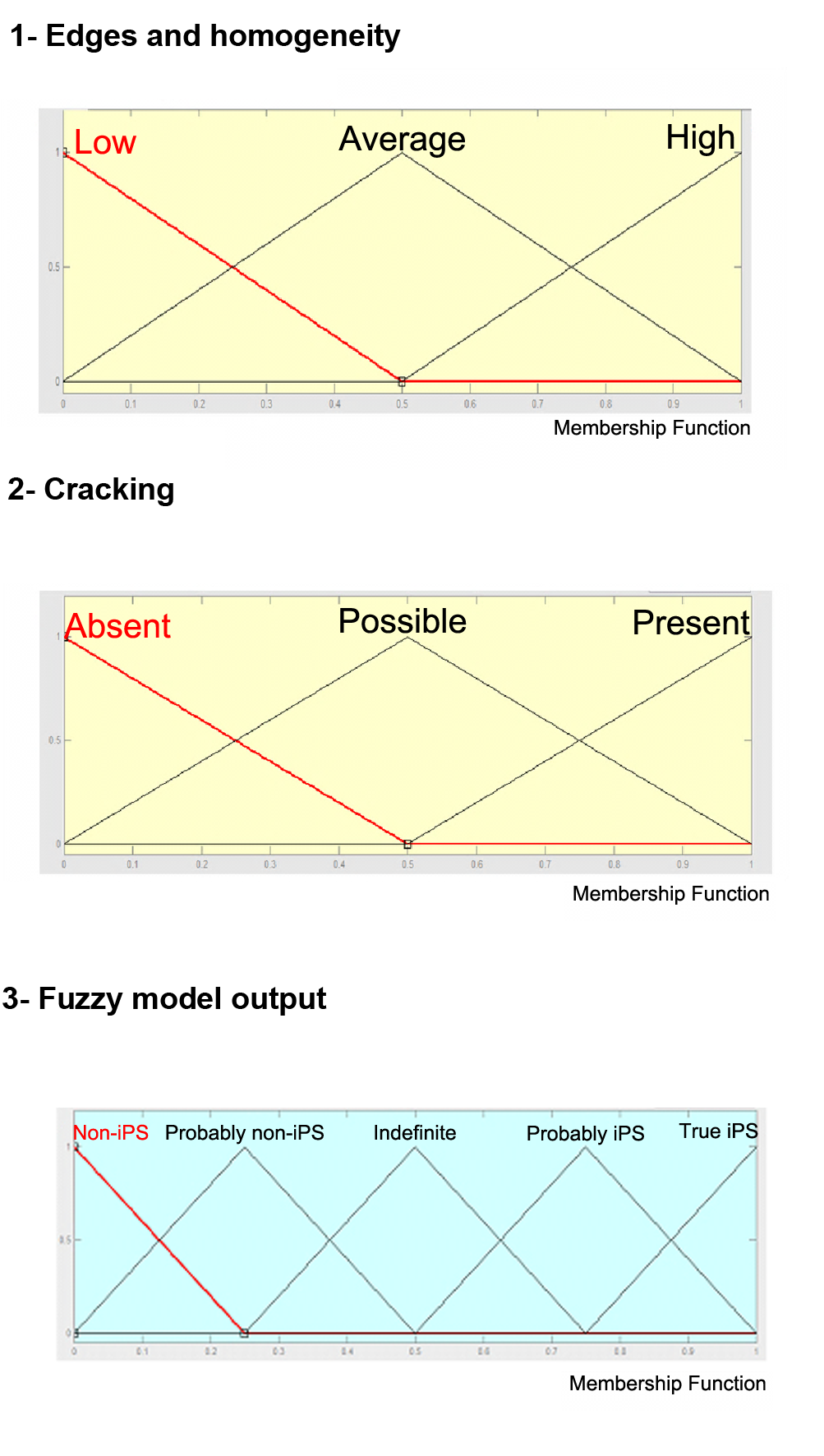

Supplement: Figure S2 — Membership functions of the fuzzy sets. The scores for colony's (A) border definition degree and texture homogeneity degree have membership functions given by the predicates “Low” (from 0 to 0.5), “Medium” (0.0–1) and “High” (0.5–1). (B) Cracked texture has the predicates “Absent” (from 0 to 0.5), “Possible” (0.0 to 1) and “Present” (0.5 to 1). As output, after defuzzyfication, there was “not IPS cells”, “probably non-iPS”, “Undefined” “probably iPS” or “iPS” domains. The optimal threshold value to dicotomically classifies between true and false colonies were calculated as 0.434. As an example, the true iPS colony on Figure 1 can be categorized as 0.875 for texture homogeneity and border delineation and 1 for cracked texture. After the defuzzyfication, 0.72 emerges as output and the colony is classified as true. (TIF) [file pone.0070605.s002.tif]

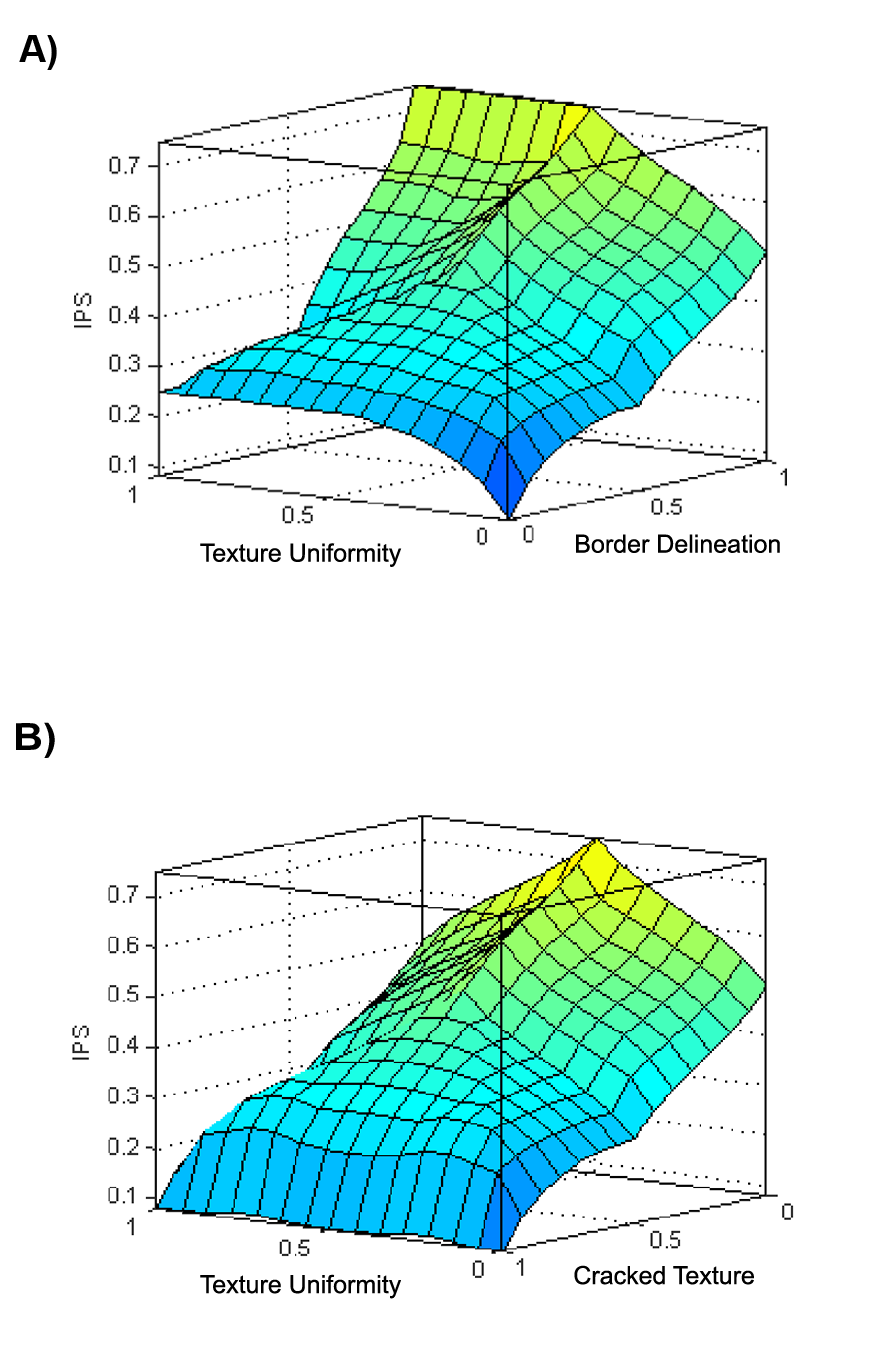

Supplement: Figure S3 — Fuzzy logic control surface output. There is a non-linear variation of the possibilities for fuzzy output depending on the observed degree of texture uniformity and (A) border delineation or (B) cracked texture. (TIF) [file pone.0070605.s003.tif]

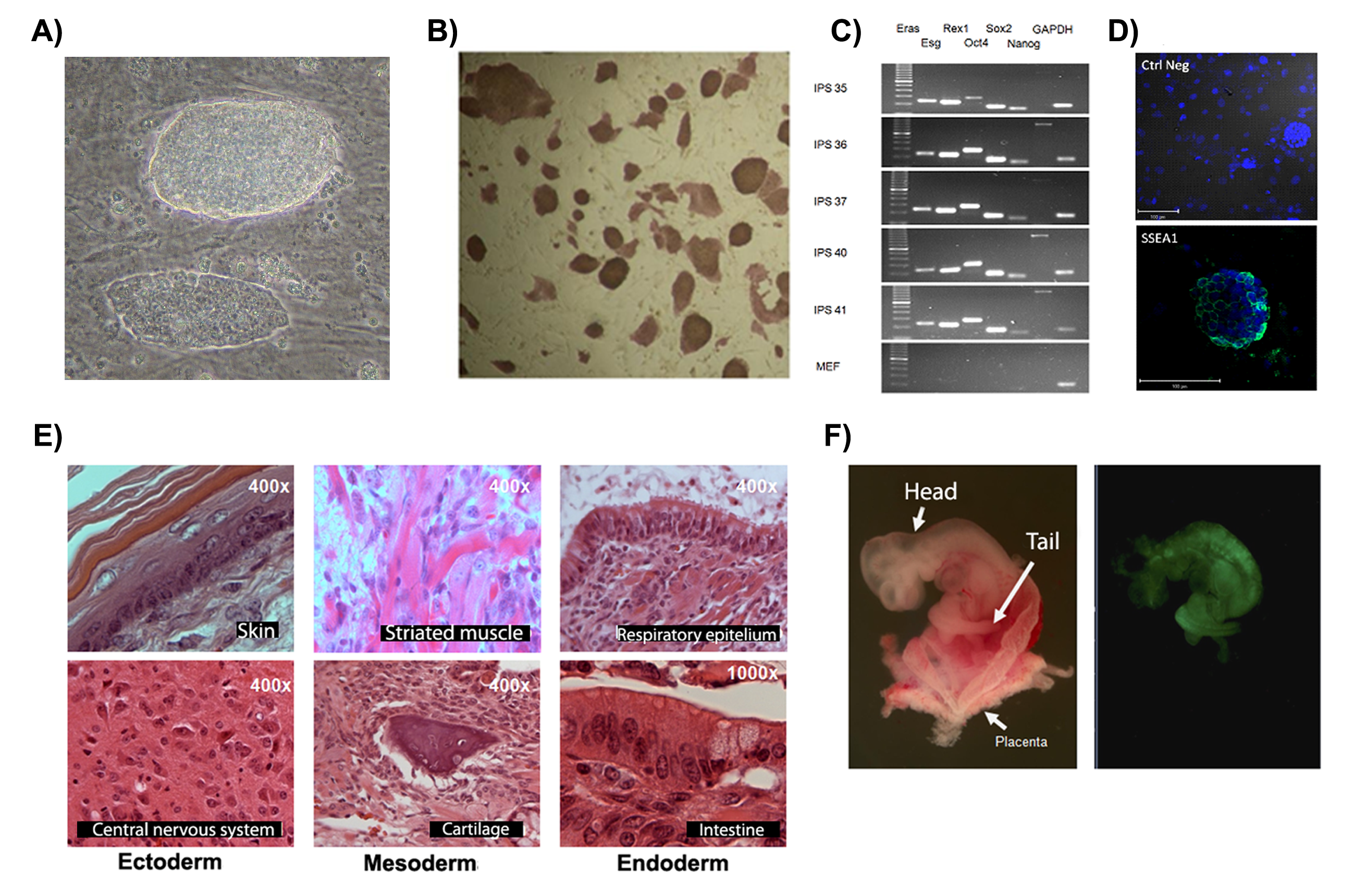

Supplement: Figure S4 — iPS generation using retrovirus. (A) Morphology of a positive iPS cell after picking (B) representative picture of alkaline phosphatase activity after picking (100x magnification). (C) Verification of pluripotency markers by RT-PCR. (D) Visualization by fluorescence microscopy of SSEA1 iPS cells marker. (E) Verification of the 3 germ layers formation on teratoma structures in nude animals. (F) Chimerical mouse fetus (in E10) viewed under 20x magnification (right filter for GFP). It is possible to see that virtually the whole body of the embryo is fluorescent, including the heart and the developing members, but not the placenta (non-iPS origin). (TIF) [file pone.0070605.s004.tif]
