# Supplementary material for: Development of a New Approach to Aid in Visual Identification of Murine iPS Colonies Using a Fuzzy Logic Decision Support System
Source: PLoS One. 2013 Aug 8;8(8):e70605. doi: 10.1371/journal.pone.0070605 (PMC3738584; doi:10.1371/journal.pone.0070605)
Supplement: Materials and Methods S1 — (DOCX) [file pone.0070605.s005.docx]

**Supplementary Materials and Methods**

## Analysis of gene expression using RT-PCR

The expression of specific markers of ES was evaluated by RT-PCR (initial denaturation for strands separation for 5 min at 95ºC followed by 28 cycles of denaturation for 15 s at 95ºC, annealing for 1 min at 60ºC, extension for 1 min at 72ºC, and final extension for 10 min at 72ºC) using specific primers. We used several genes described as important for maintaining ES cell pluripotency (Eras, Esg, Rex1 and Nanog) as well as factors used for reprogramming (Oct4 and Sox2). GAPDH was amplified in all samples (**Figure S4C**). Primers sequences used for amplifications were as follows:

**ERAS**-FW 5’ CATCCAGATGACTCACCAATGCTTCGTG 3’;

**ERAS**-RV 5’ TGGACCATATCTGCTGCAACTGGTCCAG 3’

**ESG1**-FW 5’ AATATCTGTTTGGCCCACAGGGATCTCG 3’;

**ESG1**-RV 5’ TCATGGATTCCTCCAGCTTCAGCACTC 3’;

**REX1**-FW 5’ CTGACGGATACCTAGAGTGCATCATACGAG 3’;

**REX1**-RV 5’ AGTACACACCGCCCTCTGGCTTCTCTGCAG 3’;

**OCT3/4**-FW 5’ GACAACAATGAGAACCTTCAGGAGATATGC 3’;

**OCT3/4**-RV 5’ CCAAGCTGATTGGCGATGTGAGTGATCTGC 3’;

**SOX2**-FW 5’ GTGAACCAGCGCATGGACAGCTACGCGC 3’;

**SOX2**-RV 5’ TCGTAGCGGTGCATCGGTTGCATCTGTGC 3’;

**Nanog**-FW 5’ ATGAGTGTGGGTCTTCCTGGTCCC 3’;

**Nanog**-RV5 5’ GTAAGGATCCTCATATTTCACCTGGTGGAGTCAC 3’

**GAPDH**-FW 5’ TGCACCACCAACTGCTTAG 3’

**GAPHD**-RV 5’ GATGCAGGGATGATGTTC 3’

## Immunofluorescence for SSEA-1 using confocal microscopy

iPS cells were grown in a 0.1% collagen coated glass coverslip and stained using SSEA-1 antibody (2ug/mL; Millipore Cat Num MAB4301) and Alexa 488 secundary antibodies. The images were obtained using a Zeiss LSM 510 Meta microscope using sequential excitation with leisure in 364nm and 488nm from, respectively, the Enterprise UV and argon laser. The signal sequence was collected by blue (385-460nm bypass) and green (505-555nm bypass) and emission filters analyzed by Zeiss software. White bar represents 100µm size (**Figure S4D)**.

## Teratoma formation and histological analysis

Some colonies were tested for their potential in generating teratomas with the 3 germ layers. For this purpose, 2 million iPS cells were implanted subcutaneously in the dorsal flank of nude mice acquired and maintained in the Department of Immunology of the University of São Paulo. Four weeks after injection, tumors were dissected and fixed in 10% formaldehyde. The tissues were cut and processed for histological analysis by hematoxylin and eosin staining (**Figure S4E**).

## Generation of chimeras

C57BL/6J animals injected with chorionic gonadotropin for superovulation (PMSG and HCG) and put to mate. Morula stage embryos were obtained by flushing the uterine horns after 48h. Then, after the development of embryos to the blastocyst stage in culture, they were placed in a drop of DMEM with 15% FCS under mineral oil. In a micromanipulator, with 12- to 15-µm internal diameter needle, 10 to 15 GFP^+^ iPS cells were injected into the cavity of each blastocyst. Finally, 20 blastocysts were injected into one uterine horn of each C57BL/6J female, to maximize efficiency. Females were sacrificed when embryos were at E10 and they were visualized on magnifying glass with fluorescence filter for GFP. This procedure was performed for some colonies to certify that it was possible to obtain fully pluripotent colonies (**Figure S4F**).
